# Spatio-temporal expression profile of NGF and the two-receptor system, TrkA and p75NTR, in experimental autoimmune encephalomyelitis

**DOI:** 10.1186/s12974-020-1708-9

**Published:** 2020-01-29

**Authors:** Nickoleta Delivanoglou, Marina Boziki, Paschalis Theotokis, Evangelia Kesidou, Olga Touloumi, Nikolina Dafi, Evangelia Nousiopoulou, Roza Lagoudaki, Nikolaos Grigoriadis, Ioannis Charalampopoulos, Constantina Simeonidou

**Affiliations:** 1 Laboratory of Experimental Neurology and Neuroimmunology, B’ Department of Neurology, AHEPA University Hospital, School of Medicine, Aristotle University of Thessaloniki, Thessaloniki, Greece; 2 Laboratory of Experimental Physiology, Department of Physiology and Pharmacology, School of Medicine, Aristotle University of Thessaloniki, Thessaloniki, Greece; 3 Laboratory of Pharmacology, Department of Basic Sciences, School of Medicine, University of Crete, Heraklion, Greece; 4 Institute of Molecular Biology and Biotechnology, Foundation of Research and Technology Hellas, Heraklion, Greece

**Keywords:** NGF, TrkA, p75NTR, EAE, Neuroinflammation

## Abstract

**Background:**

Nerve growth factor (NGF) and its receptors, tropomyosin receptor kinase A (TrkA) and pan-neurotrophin receptor p75 (p75NTR), are known to play bidirectional roles between the immune and nervous system. There are only few studies with inconclusive results concerning the expression pattern and role of NGF, TrkA, and p75NTR (NGF system) under the neuroinflammatory conditions in multiple sclerosis (MS) and its mouse model, the experimental autoimmune encephalomyelitis (EAE). The aim of this study is to investigate the temporal expression in different cell types of NGF system in the central nervous system (CNS) during the EAE course.

**Methods:**

EAE was induced in C57BL/6 mice 6–8 weeks old. CNS tissue samples were collected on specific time points: day 10 (D10), days 20–22 (acute phase), and day 50 (chronic phase), compared to controls. Real-time PCR, Western Blot, histochemistry, and immunofluorescence were performed throughout the disease course for the detection of the spatio-temporal expression of the NGF system.

**Results:**

Our findings suggest that both NGF and its receptors, TrkA and p75NTR, are upregulated during acute and chronic phase of the EAE model in the inflammatory lesions in the spinal cord. NGF and its receptors were co-localized with NeuN^+^ cells, GAP-43^+^ axons, GFAP^+^ cells, Arginase1^+^ cells, and Mac3^+^ cells. Furthermore, TrkA and p75NTR were sparsely detected on CNPase^+^ cells within the inflammatory lesion. Of high importance is our observation that despite EAE being a T-mediated disease, only NGF and p75NTR were shown to be expressed by B lymphocytes (B220^+^ cells) and no expression on T lymphocytes was noticed.

**Conclusion:**

Our results indicate that the components of the NGF system are subjected to differential regulation during the EAE disease course. The expression pattern of NGF, TrkA, and p75NTR is described in detail, suggesting possible functional roles in neuroprotection, neuroregeneration, and remyelination by direct and indirect effects on the components of the immune system.

## Background

Neuroinflammation is a characteristic reaction during central nervous system (CNS) trauma and/or disease. In addition to the classical paradigm of neuroinflammation representing a devastating process for the CNS, accumulating evidence support its beneficial side and even neuroprotective action [1–6]. As far as it concerns the autoimmune diseases such as multiple sclerosis (MS), the related mechanisms are more complicated since autoimmunity involves the orchestrated attack of the peripheral immune system to the CNS [7]. So, it is of high importance the existence of an endogenous neuroprotective mechanism to maintain the balance inside the CNS, to prevent, limit, and potentially reverse the damage [8–10]. Preserving homeostasis is one of the common characteristic of the nervous and immune system, and this could be achieved only by their close interaction and synergistic actions.

When we consider about CNS homeostasis and neuroprotection, at the top of the list are the neurotrophic factors that mediate their actions through membrane receptors. Nerve growth factor (NGF) is the prototype member of the neurotrophin family which was described by Levi-Montalcini [11]. Neurotrophin family also includes the brain-derived neurotrophic factor (BDNF), neurotrophin-3 (NT-3), and neurotrophin-4 (NT4) [12]. NGF by binding to two types of membrane receptors, TrkA and p75NTR, activates downstream signaling cascades [13]. TrkA is considered the high-affinity receptor and binds selectively the NGF, conveying pro-survival signals [14]. p75NTR belongs to the tumor necrosis factor receptor superfamily (TNFR) and binds to all mature neurotrophins with the same affinity, while being considered the high-affinity receptor for the immature isoforms of the neurotrophins, pro-neurotrophins, propagating—mainly but not exclusively—cell death [15]. p75NTR forms complex with various other receptors thus mediating a vast number of different and even opposing functions that depended on the cellular and environmental context each time [16]. When TrkA and p75NTR are co-expressed, they constitute a two-receptor heterotetrameric system that binds with high affinity to NGF and activates different pathways [17, 18]. NGF system signaling mediates neurite outgrowth, regulates sodium channel function, exerts prosurvival effects, and controls cell proliferation and differentiation [19–24].

The implication of NGF and its receptors, TrkA and p75NTR (from now on referred to as the NGF system), in CNS autoimmune neuroinflammation is not fully elucidated. In the attempt to clarify the role of the NGF system in autoimmunity, the animal model of MS, the EAE model, has been employed, but due to limit number of such studies, the results are quite controversial [25–31]. In the present study, we focus on the protein detection, to describe the temporal expression of the NGF system, and we associate our findings with cell-specific expression patterns under the neuroinflammatory conditions of the EAE.

## Materials and methods

### Animals

Female C57BL6/J mice (*n* = 46), 6–8 weeks old, were purchased from Hellenic Pasteur Institute (Athens, Greece). Animals were housed at the pathogen-free animal house located at Laboratory of Experimental Neurology and Neuroimmunology, B’ Neurology Department, AHEPA University Hospital, Aristotle University of Thessaloniki (Thessaloniki, Greece). They were taken care of by authorized personnel according to the University guidelines and in compliance with the Directive 2010/63/EU of the European Parliament and the Council of the European Union. The mice were provided with standard chow diet and water ad libitum. All the experimental procedures were approved by the Directorate General for Agricultural Economics and Veterinary, Directorate of Veterinary Medicine, Region of Central Macedonia (No. Prot. 164556/1450) and by the Bioethics and Ethics Committee of Aristotle University of Thessaloniki (Νo. Prot. 392).

### Induction of the EAE model

The EAE model was induced as previously described [32, 33]. Briefly, on the induction day (D0), the mice were inoculated subcutaneously, at the paralumbar area, with 300 μg of myelin oligodendrocyte glycoprotein 35-55 peptide (ΜOG_35-55_ peptide) (generously provided by Prof. Matsoukas, University of Patras, Greece) emulsified in 100 μl phosphate-buffered saline (PBS) with equal volume of complete Freund’s adjuvant (CFA), which consists of incomplete Freund’s adjuvant (IFA) enriched with 4 mg/ml *Mycobacterium tuberculosis* (strain H37Ra, MT, Thermo Fischer Scientific, Haverhill, MA, USA). A repeated same-dose injection was performed at D7 (7 days after the first injection at D0). In addition, on D0, mice were injected intraperitoneally (i.p.) with 400 ng of pertussis toxin (Sigma-Aldrich GmbH, Germany) reconstituted in 500 μl PBS buffer, and a repeated half-dose injection was performed after 2 days (D2). A number of animals, on the same days, were inoculated with CFA only and served as the control group (*n* = 10). The evaluation of the clinical course of the EAE model was daily recording of the animals weight (in grams) and their grade of paralysis based on the following scale: 0, asymptomatic; 1, partial loss of tail tonicity; 2, flaccid tail paralysis; 3, difficulty to reverse on four limbs from a supine position; 4, hindlimb paralysis; 5, forelimb paresis; and 6, death caused by EAE.

### Study time points and tissue processing

Mice were euthanized at specific time points during the disease course such as the following: at D10, the 10th day post-immunization (*n* = 8), at the acute phase (days 18–22 post-immunization) (*n* = 12), and at the chronic phase of the EAE model (day 50 post-immunization) (*n* = 14). Euthanization was conducted in a humanly way depending on further tissue processing. For the molecular techniques, the mice were deeply anesthetized and decapitated. The CNS tissues, brain and spinal cord, were rapidly isolated, snap frozen in liquid nitrogen, and stored at − 80 °C until further processing. For the histochemical evaluation, the mice during deep anesthesia were transcardially perfused with (PBS) followed by ice-cold 4% paraformaldehyde (PFA) solution in PBS. The isolated tissues were post-fixed in 4% PFA for 16–24 h at 4 °C and further processed for paraffin-embedded sectioning at six micrometers (μm).

### Evaluation of the EAE clinical manifestation by histochemical analysis of the cell infiltration and the demyelination degree during disease course

Cell infiltration was assessed by hematoxylin-eosin (H-E) staining on 6-μm paraffin-embedded brain and spinal cord sections. The sections were mounted after ethanol dehydration, and the images were taken using the × 40 field of Zeiss conventional (bright-field) microscope. Eight to ten, 348 × 261 regions of the brain and the spinal cord section of each animal were photographed, and they were further analyzed by Fiji software. The results were expressed as cells per square millimeter (cells/mm^2^).

Demyelination degree was evaluated by Luxol Fast Blue (LBF) histochemical staining on six-μm paraffin-embedded brain and spinal cord sections. Briefly, after de-paraffinization and hydration, the sections were incubated with LFB solution overnight at 37 °C and the following day were counterstained with Nuclear Fast Red. Images were taken using the × 20 field of Zeiss conventional (bright-field) microscope. Eight to ten, 671 × 503 regions of the brain and the spinal cord section of each animal were photographed, and they were further analyzed by Fiji software. The results were expressed as percentage of demyelinated area/white matter area.

### Real-time PCR analysis

NGF, TrkA, and p75NTR real-time PCR (qRT-PCR) was performed in total RNA samples of the brain and spinal cord tissues. Briefly, snap frozen samples were homogenized and RNA was extracted using Trizol reagent (Invitrogen) according to manufacturer’s protocol. Complementary DNA (cDNA) was synthesized using iScript cDNA Synthesis Kit (Biorad). All reactions were performed using the 2x Master Mix SYBR (Biorad), and the results were detected by using the iQ5 I Cycler Multicolor Detection System (Biorad). β-actin was used to standardized relative expression of the target molecules. Each 20 μl total volume reaction included the following: 10 μl of the master mix, 0.7 μl of p75NTR, NGF, and β-actin and 0.9 μl for TrkA of each primer (10 pmol), 1 μl of cDNA, and nuclease free water up to 20 μl. Target gene primers are as follows: p75NTR, forward 5′ CTGCTGCTTCTAGGGGTGTC 3′ and reverse 5′ ACACAGGGAGCGGACATACT 3′ giving rise to a 248 bp product; TrkA, forward 5′ CTCGCCAGTGGACGGTAAC3′ and reverse 5′ CCTGTCTCCTCGTTTAAACC 3′ giving rise to a 162 bp product; and NGF, forward 5′ CCGCAGTGAGGTGCATAGC 3′ and reverse 5′ TAAGGGAACTGTGTCGGGAG 3′ giving rise to a 166 bp product. p75NTR was amplified for 40 cycles (94 °C for 30 s, 60 °C for 15 s, 72 °C for 30 s, and 72 °C for 5 min), whereas TrkA and NGF cDNA were amplified for 40 cycles (94 °C for 30 s, 60 °C for 30 s, 72 °C for 30 s, and 72 °C for 5 min). Relative gene expression was calculated using the 2−ΔΔCt method. The p75NTR/TrkA ratio was deducted from the relative gene expression of each receptor.

### Western blot analysis

Western blotting for NGF, TrkA, and p75NTR was performed in total protein samples of the brain and spinal cord tissues. Briefly, snap frozen samples were homogenized in ice-cold lysis buffer consisting of 10 mM Hepes pH 7.4, 10 mM KCL, 0.1 mM EDTA, 10% NP-40, 0.1 mM EGTA, 1 mM DTT, and a mixture of protease inhibitors (1 mM PMSF, 1 μg/ml leupeptin, 1 μg/ml aprotinin). The total protein concentration was assessed by Bradford method. Twenty micrograms of protein lysates were resolved on SDS-PAGE in 8% and 10% acrylamide gel and transferred onto polyvinylidene difluoride (PVDF) membranes (Macherey-Nagel, GmbH&Co, Germany). The membranes were blocked with 5% BSA in TBS supplemented with 0.1% Tween-20 (P1379, Sigma) (TBST) for 1 h at room temperature (RT), followed by overnight incubation at 4 °C with primary antibodies such as p75NTR (1:100, sc-271708, Santa Cruz Biotechnology, Dallas, TX, USA and 1:700 anti-CD271, Biolegend), NGF (1:250, AB1526, Millipore), and TrkA (1:250, sc-118, Santa Cruz Biotechnology, Dallas, TX, USA). The membranes were washed three times for 10 min with TBST, incubated with the appropriate HRP-conjugated secondary antibodies secondary (anti-mouse 1:1.000, HRP-conjugated anti-mouse IgG (Cell Signaling Technology, Leiden, The Netherlands) and anti-rabbit 1:10.000,) for 1 h at RT and then developed using the chemiluminescence reaction (ECL, GenScript, NJ, USA). All membranes were stripped and re-probed with β-actin as loading control. Image analysis and quantification of bands’ density were performed with the software Fiji. The p75NTR/TrkA ratio was calculated from the normalized values from optical density measurements.

### Immunohistochemical spatio-temporal detection of NGF, TrkA, and p75NTR in the CNS

Protein expression on tissue was evaluated by histochemistry for NGF, TrkA, and p75NTR. Briefly, 6-μm sections were de-paraffinized, hydrated, and blocked with peroxidase in methanol for minimizing the endogenous peroxidase reaction. Antigen retrieval was performed with Tris buffer pH = 6.0, and blocking solution was applied on sections for 30 min consisting of 10% fetal bovine serum (FBS) (50115, Millipore) in 1× PBS containing, where it was necessary, 2% normal goat serum (NGS) (GO-605/100, Biosera) and 0.3% Triton-X (56,029, Reidel-de Haen). The sections were incubated overnight at 4 °C with the primary antibodies raised against NGF (1:100, AB1526, Millipore), TrkA (1:100, 763, sc-118, Santa Cruz Biotechnology, Dallas, TX, USA), and p75NTR (see below) and rinsed with TBS followed by incubation with the appropriate secondary antibodies for 1 h at RT (goat anti-rabbit IgG (Vector Laboratories, Burlingane, CA, USA, anti-mouse, Santa-Cruz) and also incubation with avidin-peroxidase (A3151, Sigma) for 1 h at RT. Positive cells were visualized as brown using the chromogenic 3,3′-diaminobenzidine (DAB, Fluka, Honeywell, Mexico City, Mexico) and counterstained with hematoxylin (Merck Millipore, Billerica, MA, USA). Images were taken using the × 40 field of Zeiss conventional (bright-field) microscope, and for each animal, 8–10, 348 × 261 regions were used to count the positive expressing cells for each target molecule against the total cell number of the selected region. The results were expressed as cells per square millimeter (cells/mm^2^).

Three different antibodies were used for p75NTR detection: rabbit anti-Human p75NTR, Promega G2301, mouse NGFR p75 (B-1): sc-271708, Santa-Cruz, and rabbit Anti-CD271, p75NTR, Biolegend. The two latter antibodies were tested and exhibited the same regional and cellular expression pattern with this of Promega.

### Localization and neuropathological evaluation of the target molecules NGF, TrkA, and p75NTR by immunofluorescence

For identifying the type of the positive cells for the target molecules, double-immunofluorescent staining was undertaken. Briefly, after deparaffinization, hydration, and antigen retrieval using a Tris-EDTA buffer pH = 8.5, sections were incubated overnight with a combination of each of the target molecules NGF, TrkA, and p75NTR with the following antibodies: NeuN for mature neurons (mouse, MAB377, Millipore), SMI32 for degenerated axons (mouse, NE1023, Calbiochem), GAP43 for regenerating axons (mouse, GAP-7B10, Sigma), CNPase for oligodendrocytes (mouse, MAB326, Millipore), GFAP for astrocytes (mouse, G9264, DAKO, Agilent Technologies, Santa Clara, CA, USA), arginase I for M2 phenotype microglia (goat, sc-18354, Santa-Cruz), inducible nitric oxide synthase (iNOS/NOS2) for M1 phenotype microglia (mouse, sc-7271, Santa-Cruz), Mac-3 for macrophages (rat), CD3 for T lymphocytes (rabbit, RM-9107-SO, Thermo Scientific, and mouse, sc-20047, Santa-Cruz), and B220 for B lymphocytes (CD45R/B220 rat, 550286, BD). Secondary antibodies were applied depending on the first antibodies as follows: goat anti-rabbit (CF 555, 20033,Biotium, Fremont, CA, USA), chicken anti-rabbit (CF 488A, 20209, Biotium, Fremont, CA, USA), goat anti-rabbit (568, AlexaFluor, Thermo Fischer Scientific, Haverhill, MA, USA), goat anti-mouse (CF 488A, 20010, Biotium, Fremont, CA, USA), goat anti-mouse (CF 555, 20030, Biotium, Fremont, CA, USA), donkey anti-goat (CF 555, 20039, Biotium, Fremont, CA, USA), and goat anti-rat (CF 555, 20233, Biotium, Fremont, CA, USA). Sections were mounted with Hoechst antifade reagent which counterstains nuclei. Images were taken using the × 40 field of Zeiss fluorescent microscope and Nikon C1-Eclipse TE-2000U confocal microscope. For each animal, 8–10, 348 × 261 regions were used to count the positive cells for each marker against the total number of cells positive for each target molecule of the selected region. The results were expressed as cells per square millimeter (cells/mm^2^). For GAP43, measurements were expressed as integrated density using the Fiji software. The levels of co-localization were reported as percentage, and they were counted as the number of the double-positive cells against the total positive cells multiplied by 100. For GAP-43, the levels were calculated as integrated density of the double-positive region against the total positive region multiplied by 100.

### Statistical analysis

Data analysis was performed using GraphPad Prism 7.0 and SPSS 18.0 software. More specifically, for normality distribution assess, Kolmogorov-Smirnov and Shapiro-Wilk tests were used. The differences between experimental groups were assessed with one-way analysis of variance and post hoc Bonferroni’s test for parametric data and with the Kruskal-Wallis and Dunn’s post hoc for non-parametric data. All values are expressed as mean ± standard error mean (SEM), and the differences were taken into account as statistically significant when *p* < 0.05.

## Results

### Assessment of the EAE clinical course, the cell infiltration, and the demyelinating degree

**Fig. 1:**
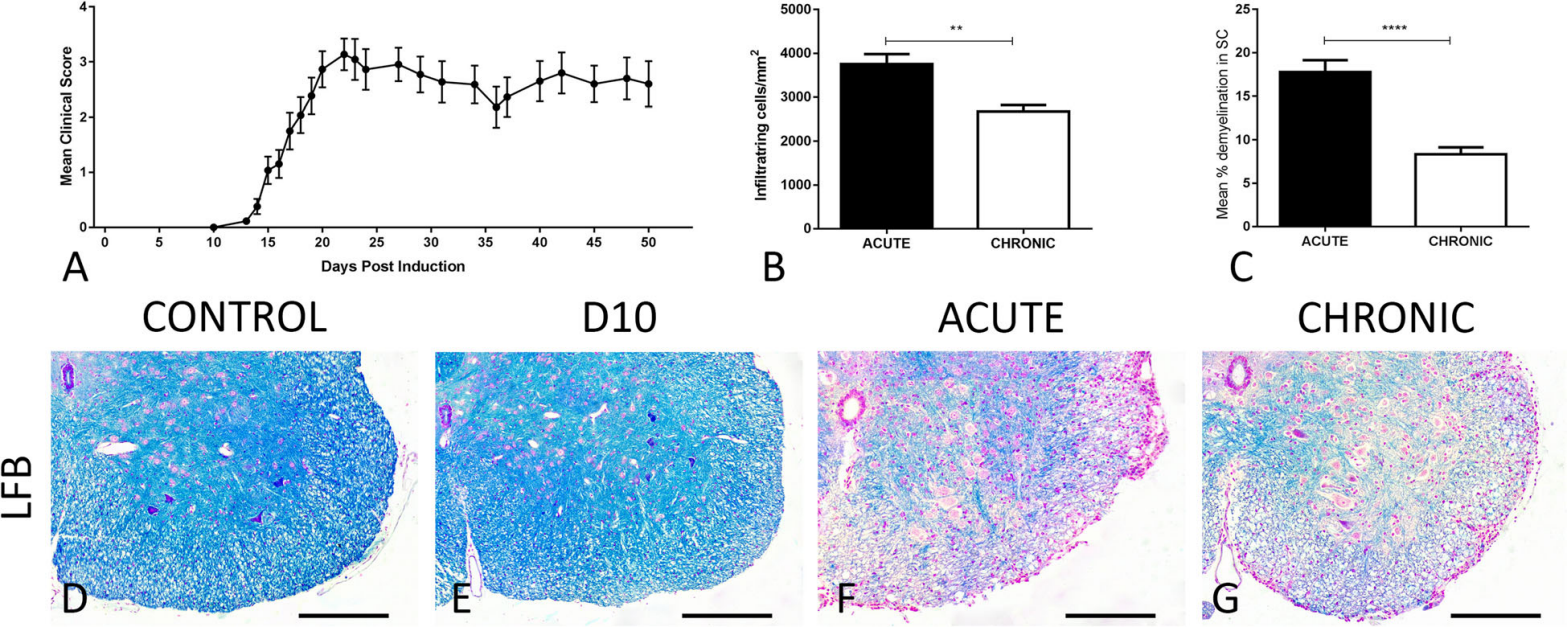
**a** The mean clinical score of mice during the MOG-induced EAE model. **b** The graphical representation of the infiltrates (cells/mm^2^) during the acute and chronic phase of the EAE model. **c** Graphical representation of the percentage of demyelinating degree during the EAE course. **d**–**g** Images of the Luxol Fast Blue (LFB) staining for evaluation of the demyelination degree among the groups (control, D10, acute, and chronic) of the EAE model. *p < 0.05, **p < 0.01, ***p < 0.001, ****p < 0.0001. Scale bar = 100 μm

Mice inoculated with the MOG_35-55_ peptide developed a moderate to severe chronic course of EAE model, with no observed mortality (MMS = 347.5 ± 0.31 and AUC = 91.5 ± 7.5) (Fig. 1a). Mice inoculated with CFA did not develop EAE, and they served as the control group. We focused our study on the spinal cord which exhibits the greatest degree of the inflammatory response, in contrast to the brain in which the inflammation is limited if not observed at all (data not shown) [34–36]. The cells inside the inflammatory loci during the acute phase (3754 ± 229.5 cells/mm^2^) outnumbered the cells observed during the chronic phase (2680 ± 142 cells/mm^2^) of the EAE disease course (Fig. 1b). In addition, a high demyelinating degree was observed at the acute (17.7 ± 1.36) compared to the chronic phase (8.3 ± 0.8) by LFB staining (Fig. 1c–g).

### Analysis of NGF, TrkA, and p75NTR mRNA expression in the CNS by qRT-PCR

**Fig. 2:**
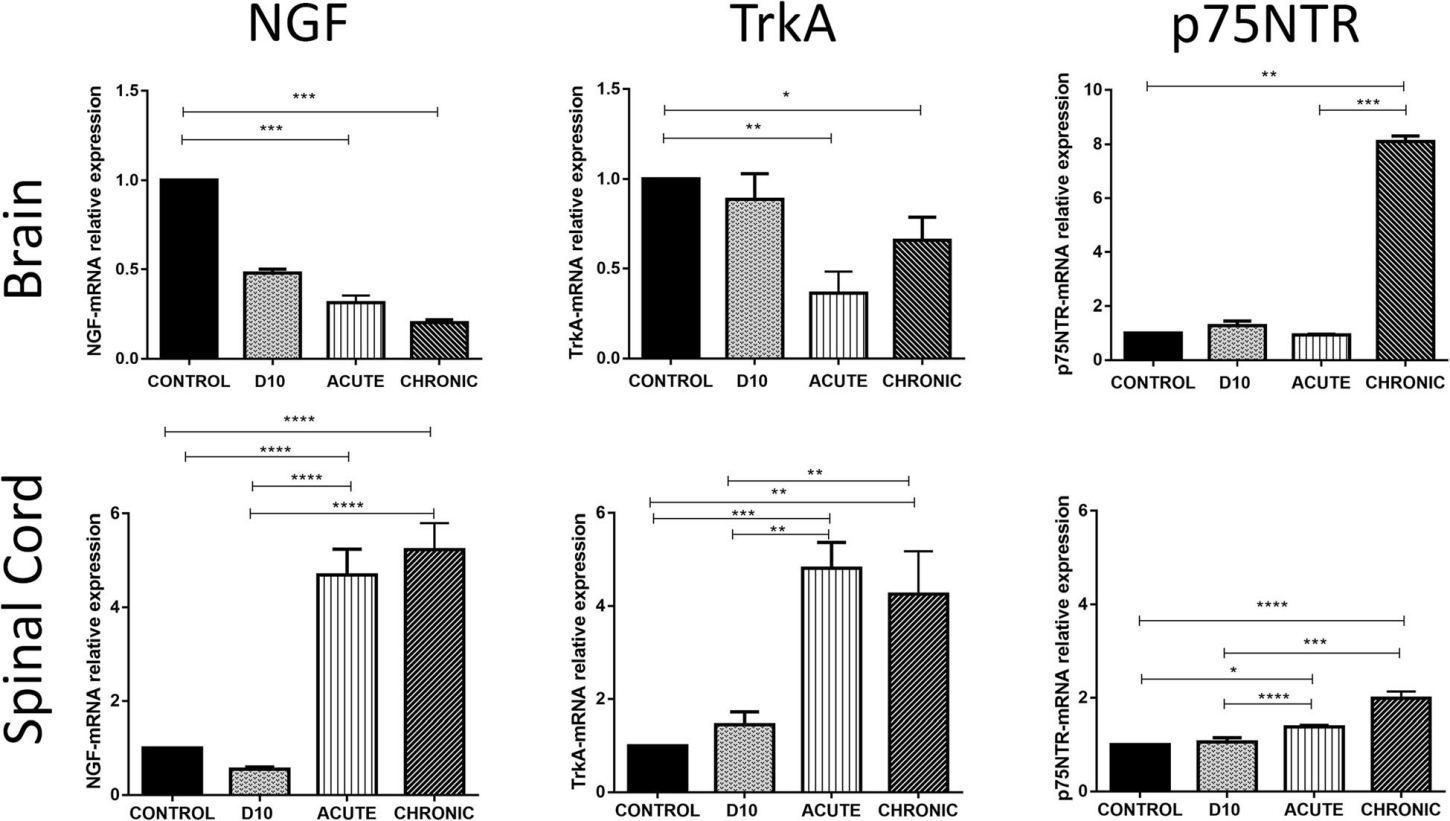
mRNA relative expression of NGF and its receptors TrkA and p75NTR by qPCR analysis of brain (upper panel) and spinal cord (lower panel) samples during EAE course. *p < 0.05, **p < 0.01, ***p < 0.001, ****p < 0.0001

The mRNA levels of NGF and its receptors were analyzed in the brain and in the spinal cord of all the groups (Fig. 2 upper and lower panel, respectively). In the brain, NGF-mRNA levels were significantly decreased at the acute (0.3-fold) and the chronic phase (0.2-fold) compared to control group (*p* = 0.0015 and *p* < 0.001 respectively). The same reduced expression pattern was observed for TrkA-mRNA reaching the lowest levels at the acute phase (0.36-fold), and they remained decreased during the chronic phase (0.66-fold) of the EAE model (*p* = 0.0019 and *p* = 0.0429). On the contrary, p75NTR-mRNA level was elevated during the chronic phase (8-fold) (*p* = 0.0048). Additionally, a sub-analysis of the p75NTR to TrkA ratio exhibited a robust increase at the chronic phase of the EAE model (13-fold) (*p* < 0.0001) (Additional file 1: Figure S1A). In the spinal cord, NGF, TrkA, and p75NTR mRNAs were highly expressed during the two phases compared to control animals. More precisely, NGF-mRNA at the acute phase was increased (4.7-fold) (*p* < 0.001) and at the chronic (5.2-fold) (*p* < 0.0001). TrkA-mRNA exhibited upregulated expression at the acute (4.8-fold) (*p* = 0.002) and at the chronic phase (4.3-fold) (*p* = 0.0016). Also, p75NTR-mRNA showed an increased expression at the acute and chronic phase ((1.4-fold) (*p* = 0.0234) and (2-fold) (*p* < 0.0001) respectively). p75NTR to TrkA ratio was found decreased at the acute and the chronic phase compared to control group (0.3-fold, *p* < 0.0001, and 0.5-fold, *p* = 0.0034, respectively) (Additional file 1: Figure S1B).

### Analysis of NGF, TrkA, and p75NTR total protein expression in the CNS by Western blotting

**Fig. 3:**
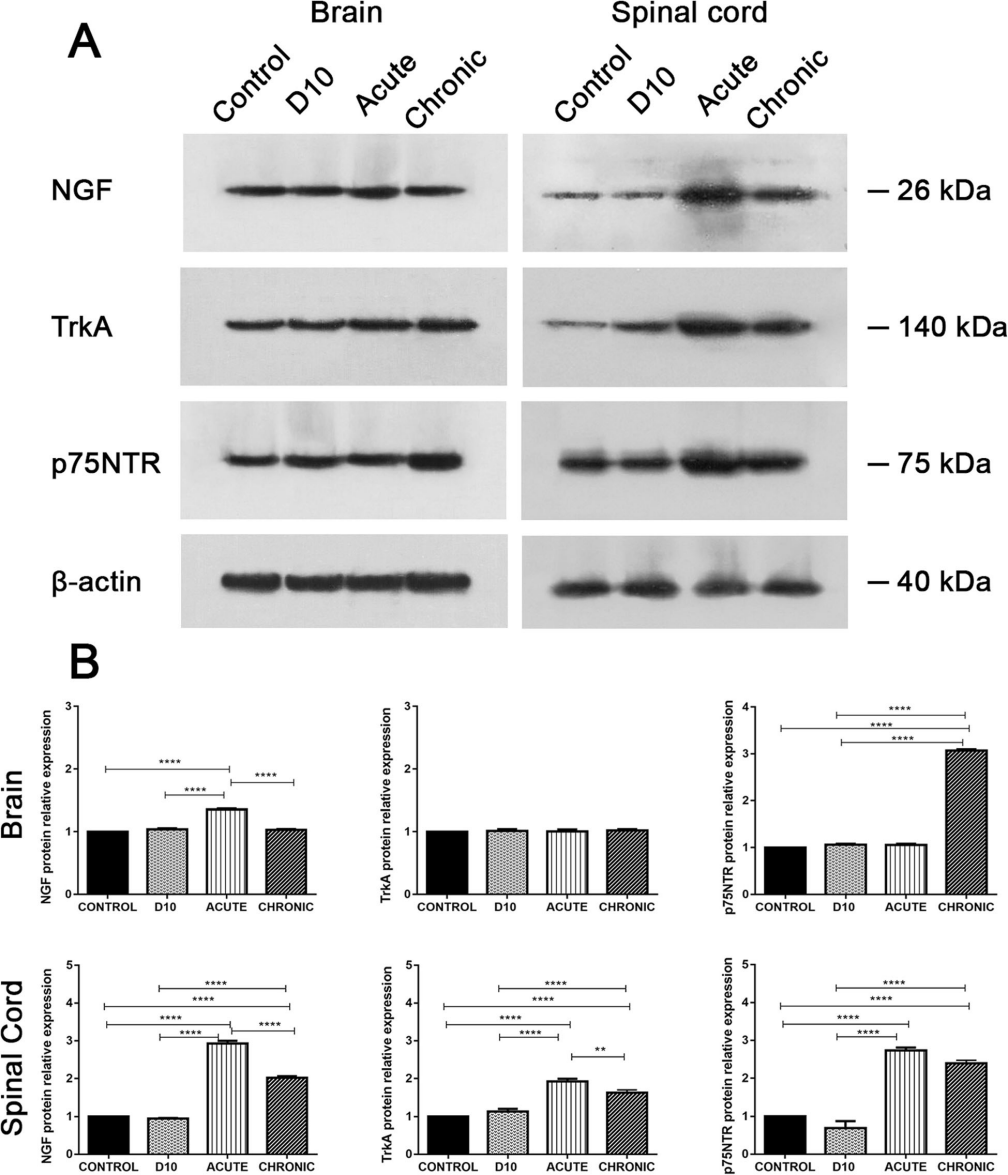
**a** Representative images from developed membranes of the NGF, TrkA, and p75NTR proteins by Western blotting analysis of brain (left panel) and spinal cord samples (right panel). **b** The respective graphical representation of their relative expression in the brain (upper panel) and spinal cord (lower panel). All samples were standardized to β-actin which was used as loading control. *p < 0.05, **p < 0.01, ***p < 0.001, ****p < 0.0001

Analysis of the total brain and spinal cord lysate samples by Western blot for NGF and its receptors revealed a differentiated expression pattern during the acute and chronic phase compared to control group, as it is shown in the gel images (Fig. 3a) and their respective graphical representation (Fig. 3b).

In the brain, NGF protein expression was slightly increased during the acute phase (1.4-fold) compared to control group, whereas TrkA expression was stable and consistent between the groups. As far as it concerns, the p75NTR protein expression was upregulated during the chronic phase (3-fold) (Fig. 3b, upper panel). The p75NTR to TrkA ratio analysis showed a considerable increase during the chronic phase (3-fold) (*p* < 0.0001) (Additional file 2: Figure S2A).

The spinal cord sample analysis showed for NGF an upregulated expression during the acute (3-fold) and the chronic phase (2.8-fold). TrkA showed a gradual increase at the D10 (1.3-fold), at the acute (1.9-fold), and at the chronic phase (1.6-fold) compared to control group. As far as it concerns, the p75NTR was increased during the acute and the chronic phase (2.7-fold and 2.3-fold, respectively) (Fig. 3b, lower panel). p75NTR to TrkA ratio was found slightly increased compared to control group at the acute and chronic phase of the EAE (1.45-fold, *p* < 0.0001, and 1.5-fold, *p* < 0.0001, respectively) (Additional file 2: Figure S2B).

### Protein expression of the NGF and its receptors in the white and grey matter of the spinal cord

**Fig. 4:**
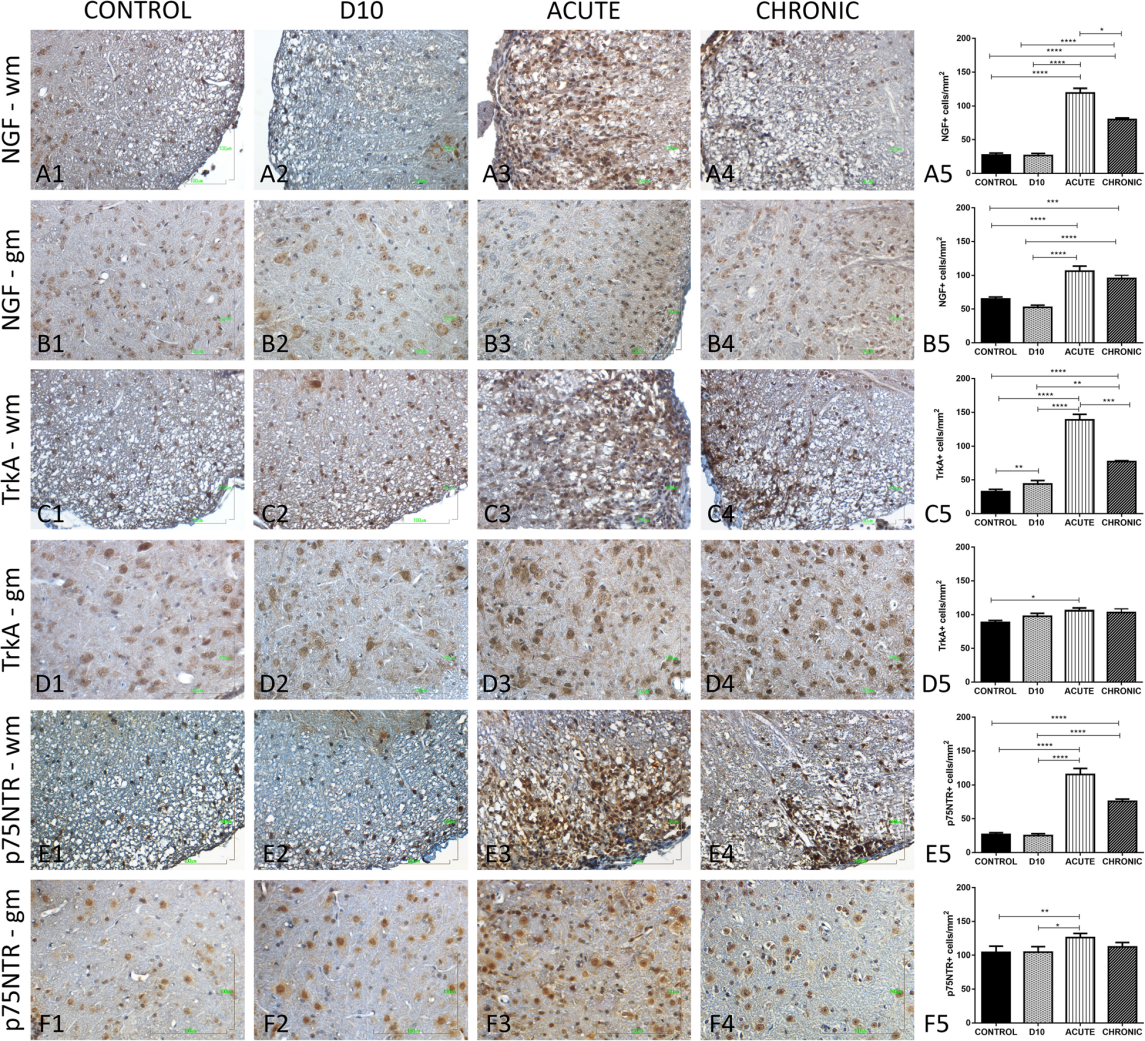
Mapping of the expression of NGF and its receptors, TrkA and p75NTR, by histochemical analysis of the spinal cord tissue during the EAE course. **a1**–**a5** Images of NGF expression in the white matter of the spinal cord among the groups of the EAE model and its representative graph. **b1**–**b**5 Images of NGF expression in the grey matter of the spinal cord among the groups of the EAE model and its representative graph. **c1**–**c**5 Images of TrkA expression in the white matter of the spinal cord among the groups of the EAE model and its graphical representation. **d1**–**d**5 Images of TrkA expression in the grey matter of the spinal cord among the groups of the EAE model and its graphical representation. **e1**–**e**5 Images of p75NTR expression in the white matter of the spinal cord among the groups of the EAE model and its representative graph. **f1**–**f**5 Images of p75NTR expression in the grey matter of the spinal cord among the groups of the EAE model and its representative graph. *p < 0.05, **p < 0.01, ***p < 0.001, ****p < 0.0001. Scale bar = 100 μm

As it is mentioned before, our investigation was targeted to the spinal cord, where we observed that in the normal appearing white matter (NAWM), the NGF system exhibited a stable expression among all the groups (data not shown). In the lesioned white matter area, the expression of the NGF system was greatly upregulated compared to the control group. More specifically, NGF protein expression is elevated during acute (119.5 ± 6.48, *p* < 0.0001) and chronic phase (79.86 ± 2.51, *p* < 0.0001) (Fig. 4a1–a5). TrkA receptor expression is upregulated during acute (138.8 ± 8.28, *p* < 0.0001) and chronic phase (77.01 ± 1.44, *p* < 0.0001), compared to control group (Fig. 4c1–c5). As well, for p75NTR, receptor expression is highly upregulated at the acute (115.1 ± 8.96, *p* < 0.0001) and chronic phase (75.9 ± 3.27, *p* < 0.0001) of the EAE course compared to control group (Fig. 4e1–e5). In the grey matter, NGF expression is elevated at acute (106.4 ± 7.45, *p* < 0.0001) and at chronic (95.55 ± 4.71, *p* < 0.0001) phase compared to control group (Fig. 4b1–b5). On the other hand, for TrkA and p75NTR, there is a slight increase at acute phase compared to control group (105.7 ± 4.1, *p* = 0.0109, and 126.1 ± 6.01, *p* = 0.0011, respectively) (Fig. 4d1–d5 and f1–f5, respectively).

### Spatial detection of NGF, TrkA, and p75NTR in the spinal cord

#### NGF analysis

**Fig. 5:**
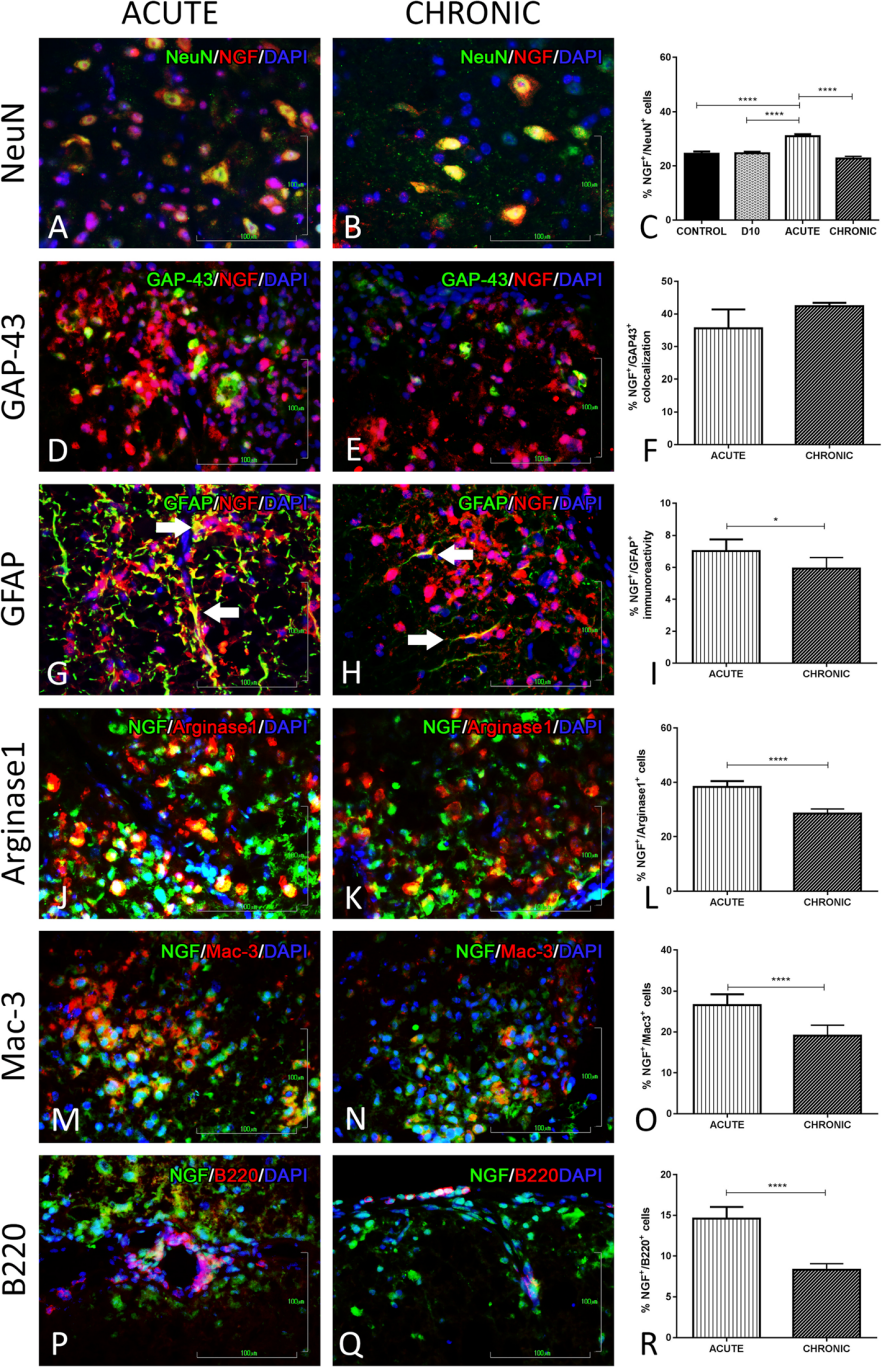
Double immunofluorescence staining of NGF expression on different cell types of the nervous and immune system. **a**, **b** DIF of NGF and NeuN in the grey matter of spinal cord at acute and chronic phase of the EAE model. **c** Graphical representation of the percentage of co-expression during the EAE course. **d**, **e** DIF of NGF and GAP-43, **g**, **h** DIF of NGF and GFAP, **j**, **k** DIF of NGF and Arginase 1, **m**, **n** DIF of NGF and Mac-3, and **p**, **q** DIF of NGF and B220, in the inflammatory loci of the white matter of the spinal cord during the acute and chronic phase of the EAE model. **f**, **i**, **l**, **o**, **r** Their graphical representations of the percentage of co-localization at the acute and chronic phase of the EAE model. *p < 0.05, **p < 0.01, ***p < 0.001, ****p < 0.0001. Scale bar = 100 μm

NGF was highly co-localized with NeuN during the acute phase of the EAE model compared to controls (31 ± 0.7, *p* < 0.0001), whereas at the chronic phase the level of co-localization is similar to control (Fig. 5a–c).

In the context of evaluating axonopathy, we studied the expression of SMI-32, a marker of degeneration and GAP-43, a marker of regeneration. Results of SMI-32 have showed that there is no immunoreactivity regarding NGF. On the other hand, for GAP-43, there was a high co-expression during acute phase with a larger percentage detected at the chronic phase for NGF (35.6 ± 5.9, *p* = 0.01, and 42.4 ± 1.03, *p* = 0.063, respectively) (Fig. 5d–f).

As far as it concerns the detection on different glial cell types, there is variable percentage of the representation in the inflammatory loci. In our EAE model, no NGF statically significant expression was found in oligodendrocytes (CNPase^+^ cells), whereas there was observed a minimal immunοreactivity with astrocytes (GFAP^+^ cells), during acute and chronic phase (7.02 ± 0.73, *p* < 0.0001, and 6 ± 0.7, *p* < 0.0001, respectively) (Fig. 5g–i). Furthermore, for microglia cells and especially type M2 (Arginase1^+^ cells), it was found that they synthesize NGF (38.3 ± 2.1, *p* < 0.0001, and 28.43 ± 1.8, *p* < 0.0001, respectively) (Fig. 5j–l). On the other hand, no co-localization with microglia cells type M1 (iNOS^+^ cells) was detected.

The immunological expression profile of NGF markers for monocytes/macrophages and for lymphocytes (T and B cells) was employed. As far as it concerns the macrophages, which are also present at the inflammatory loci during the acute and chronic phase of our experimental model of MS, they are described here for the first time to co-localize with NGF (26.6 ± 2.7, *p* < 0.0001, and 19 ± 2.6, *p* < 0.0001, respectively) (Fig. 5m–o).

As far as it concerns the mediation of lymphocytes, there is no evidence of immunoreactivity for NGF on T lymphocytes (CD3^+^ cells), whereas NGF is expressed by B lymphocytes (B220^+^ cells) in the inflammation loci during acute (14.6 ± 1.4, *p* < 0.0001) and chronic phase (8.3 ± 0.7, *p* < 0.0001) of the EAE course (Fig. 5p–r).

#### TrkA analysis

**Fig. 6:**
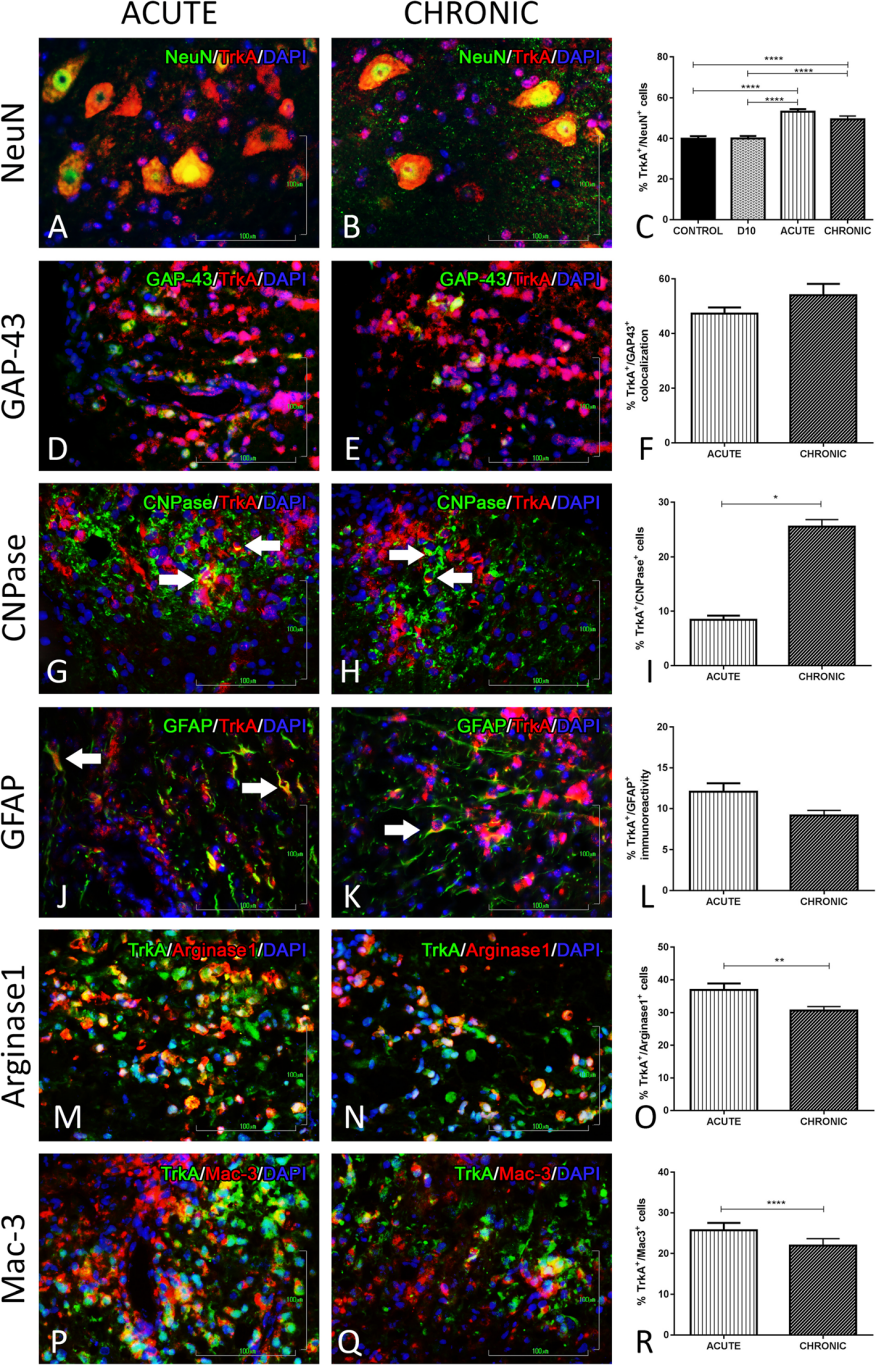
Double immunofluorescence staining of TrkA expression on different cell types of the nervous and immune system. **a**, **b** DIF of TrkA and NeuN in the grey matter of spinal cord at acute and chronic phase of the EAE model. **c** Graphical representation of the percentage of co-expression during the EAE course. **d**, **e** DIF of TrkA and GAP-43, **g**, **h** DIF of TrkA and CNPase, **j**, **k** DIF of TrkA and GFAP, **m**, **n** DIF of TrkA and Arginase1, and **p**, **q** DIF of TrkA and Mac-3, in the inflammatory loci of the white matter of the spinal cord during the acute and chronic phase of the EAE model. **f**, **i**, **l**, **o**, **r** Their graphical representations of the percentage of co-localization at the acute and chronic phase of the EAE model. *p < 0.05, **p < 0.01,*** *p* < 0.001, ****p < 0.0001. Scale bar = 100 μm

Spatial TrkA analysis has revealed a slightly higher co-localization with NeuN during acute and chronic phase compared to controls (53.2 ± 1.2, *p* < 0.0001, and 49.5 ± 1.6, *p* < 0.0001, respectively) (Fig. 6a–c). Also, there is no detected immunoreactivity with SMI-32 and TrkA, whereas there is co-expression with GAP-43 both at the acute and chronic phase where it was higher (47.3 ± 2.04, *p* = 0.0078, and 54 ± 4.2, *p* = 0.0313, respectively) compared to controls (Fig. 6d–f).

The glial expression profile evaluation yielded a differential cellular localization for TrkA during the EAE disease course. More specifically, TrkA double-positive oligodendrocytes (CNPase^+^ cells) are represented at 8.43 ± 0.77, *p* < 0.0001, during acute and at 25.5 ± 1.3, *p* < 0.0001, during chronic phase respectively (Fig. 6g–i). Astrocyte (GFAP^+^ cells) immunοreactivity with TrkA is represented at 12.7 ± 1.04, *p* < 0.0001, during acute and 9.16 ± 0.63, *p* < 0.0001, at chronic phase (Fig. 6j–l). Arginase1^+^ cells express on their surface the high-affinity receptor TrkA (37 ± 1.9, *p* < 0.0001, and 30.7 ± 1.2, *p* < 0.0001, respectively) during both the acute and the chronic phase of the EAE model (Fig. 6m–o).

The macrophages which infiltrate the CNS in the inflammatory EAE environment also express the TrkA receptor (25.7 ± 1.8, *p* < 0.0001, and 22 ± 1.7, *p* < 0.001, respectively) (Fig. 6p–r). The CD3^+^ cells and B220^+^ showed no immunoreactivity with this receptor during the EAE course.

#### p75NTR analysis

**Fig. 7:**
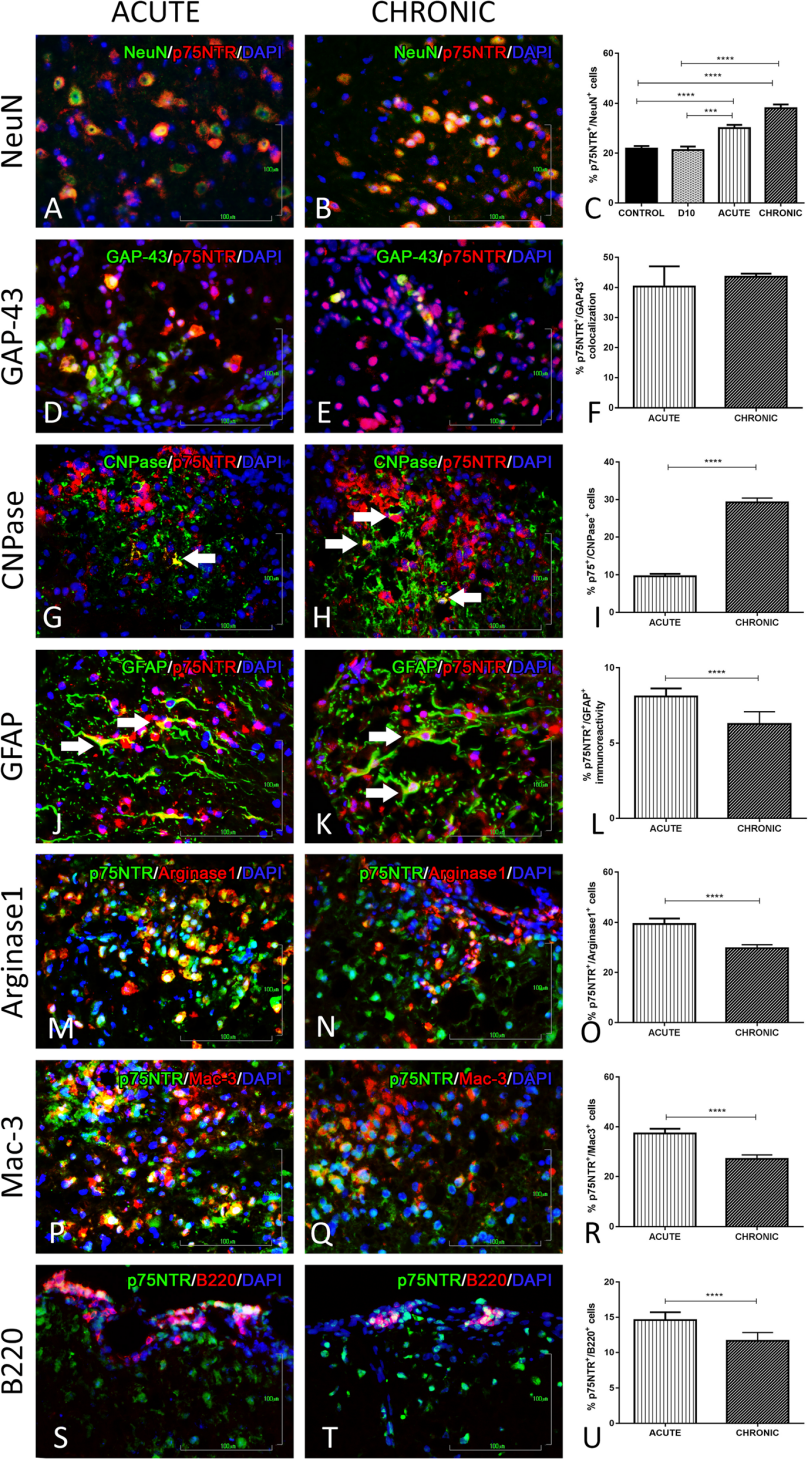
Double immunofluorescence staining of p75NTR expression on different cell types of the nervous and immune system. **a**, **b** DIF of p75NTR and NeuN in the grey matter of spinal cord at acute and chronic phase of the EAE model. **c** Graphical representation of the percentage of co-expression during the EAE course. **d**, **e** DIF of p75NTR and GAP-43, **g**, **h** DIF of p75NTR and CNPase, **j**, **k** DIF of p75NTR and GFAP, **m**, **n** DIF of p75NTR and Arginase 1, **p**, **q** DIF of p75NTR and Mac-3, and **s**, **t** DIF of p75NTR and B220, in the inflammatory loci of the white matter of the spinal cord during the acute and chronic phase of the EAE model. **f**, **i**, **l**, **o**, **r**, **u** Their graphical representations of the percentage of co-localization at the acute and chronic phase of the EAE model. *p < 0.05, **p < 0.01, ***p < 0.001, ****p < 0.0001. Scale bar = 100 μm

As far as it concerns, NeuN co-localization with p75NTR in the grey matter shows a high percentage during acute and chronic phase (30 ± 1.4, *p* < 0.0001, and 38 ± 1.6, *p* < 0.0001) compared to control group (Fig. 7a–c). Evaluation of the SMI-32 results has showed that there is no p75NTR immunoreactivity. Regarding the regenerative marker GAP-43, there was a high co-expression during acute phase and at the chronic phase where the highest level was reached compared to controls (40.3 ± 6.8, *p* = 0.0038, and 43.6 ± 1.06, *p* > 0.1000) (Fig. 7d–f).

The glial co-localization evaluation with p75NTR revealed more or less the same expression pattern with the NGF and TrkA. Specifically, p75NTR^+^/CNPase^+^ cells accounted for 9.6 ± 0.63, *p* < 0.0001, during acute and 29.3 ± 1.16, *p* < 0.0001, at chronic phase (Fig. 7g–i). p75NTR double-positive GFAP cells were estimated at 8.09 ± 0.53, *p* < 0.001, during acute phase and at 6.25 ± 0.8, *p* < 0.0001, during chronic phase of the EAE model (Fig. 7j–l). Polarized M2 microglia cells (Arginase1^+^ cells) also express the low affinity receptor p75NTR (39.4 ± 2.1, *p* < 0.0001, and 29.7 ± 1.4, *p* < 0.0001, respectively) during the two phases of the EAE disease model (Fig. 7m–o).

As far as it concerns the p75NTR expression on macrophages (Mac-3^+^ cells), it is estimated to be 37.3 ± 2, *p* < 0.0001, during the acute phase and 27 ± 1.6, *p* < 0.0001, at the chronic phase (Fig. 7p–r). In addition, even if there is no evidence of immunοreactivity with T lymphocytes (CD3^+^ cells), p75NTR receptor is expressed by B lymphocytes in the inflammation loci during acute and chronic phase (14.6 ± 1.1, *p* < 0.0001, and 11.7 ± 1.2, *p* < 0.0001, respectively) of EAE course (Fig. 7s–u).

## Discussion

During the last decades, neurotrophic factors and their receptors have emerged as key players regulating the communication between immune system and the CNS. In the present study, NGF system has been shown to be under differential expression at the specific time points of the EAE model that may regulate nervous and immune response during the inflammatory processes.

As qRT-PCR analysis has showed, the NGF and TrkA gene expression is decreased in the brain since their expression under physiological conditions is low. Moreover, there is dependence on the BDNF and its receptor TrkB, at regional and cellular level. p75NTR expression is induced under pathological conditions as at both phases of the disease course. In the inflammatory loci of the spinal cord, the expression of NGF system is highly regulated to enhance its role and downstream signaling contributing to neuroprotection.

Protein expression of the NGF system in the brain seems to maintain its levels in order to preserve and execute seamlessly its functions, especially since this tissue is not so apparent influenced from the immune attack. In the spinal cord, its levels follow the same high expression pattern during both phases of the EAE model. Our findings are in accordance with the primary function of NGF/TrkA/p75NTR system which is to promote the cellular survival under harmful conditions.

As far as it concerns the analyzed p75NTR to TrkA ratio, in the brain and spinal cord, both at gene and protein level, our observation supports that the inflammatory attack triggers selectively the NGF-mediated neuroprotection. In this respect, NGF availability matches the receptors’ expression stoichiometry that ultimately regulates the proper kinetic scheme resulting in NGF signaling [37]. Specifically, the levels of NGF and p75 to TrkA ratio exhibit the same pattern during the acute phase and in a lesser extend in chronic. In this context, some points should be taken into consideration. Firstly, the ratio is a direct depiction of the each receptor’s individual level of expression. Secondly, p75NTR can be detected as monomer, dimer, trimer, and/or oligomer exerting TrkA- and NGF-independent functions [38]. Furthermore, TrkA binds NGF and through endosomal trafficking promotes long-lasting signaling facilitating the neuroprotection [39]. Finally, the ratio analysis by both qRT-PCR and Western blot can offer a rough evaluation of the NGF’s system due to the challenging limitations of the techniques’ nature.

In our study, the experimental animals developed a severe chronic MOG-EAE manifested by immune cells infiltration and demyelination, supported also by Micera et al. [40]. We identified the expression of the NGF, TrkA, and p75NTR in the grey and white matter of the spinal cord. We observed an upregulation of the NGF synthesis and TrkA and p75NTR expression, acting as a homeostatic mechanism that potentiates the inflamed spinal cord to challenge and potentially overcome the damage. The excess of NGF synthesis and secretion further induces the higher expression of its receptors, targeting to optimize both the cellular receptiveness and the system responsiveness to the beneficial properties of the neurotrophic factor. It is noteworthy that in the NAWM, the expression of the molecules is proportionate between the different time points and it is restricted inside the lesioned area indicating the specificity of the immune response. A same observation is made in the grey matter although in a lesser extent, due to the importance of maintaining the neuron bodies and dendrites intact.

The identification of the cellular populations these molecules are expressed by is of critical importance in clarifying their role in pathogenesis and the treatment of the disease. Initially, the spatial analysis of NGF expression and its two-receptor system, TrkA and p75NTR, in the grey matter demonstrated the importance of the trophic and supportive action. The population of NeuN^+^ cells expressing NGF, TrkA, and p75NTR marked a statistically significant increase in response to the inflammatory attack. None of the molecules under investigation seems to be co-located with the SMI-32. On the other hand, the three molecules have intense immunoreactivity at GAP-43 expression sites. To our knowledge, these results are described in our study for the first time. By taking account that axonal regeneration is limited in the EAE environment, we can assume that the co-expression of NGF and the two receptors with GAP-43 is not sufficient for regeneration taking place and it is possible that the presence of other molecules is required [41]. NGF binds to p75NTR, which with the presence of TrkA forms a high-affinity complex that favors axonal elongation and cell survival including the myelinating cells [42–45]. On the contrary, in the presence of myelin debris, as in the case of inflammatory demyelination, p75NTR/NogoR/Lingo-1R complex activates RhoA leading to inhibition of the neurite outgrowth and therefore of remyelination [46–50]. Our results indicate that during the acute and chronic phase, there is myelin debris, but also cells synthesize and secrete NGF. This condition directs p75NTR towards the formation of TrkA/p75NTR complex and NGF signaling.

Regarding the expression on glial cells, our analysis showed that only a small portion of the oligodendrocyte population expresses TrkA and p75NTR located strictly within the inflammatory loci in both phases of the EAE model while there is no expression in NAWM and in the white matter of the control group. Our observation is partly supported by previous studies according to which no p75NTR expression is described in vivo under physiological conditions, whereas in the cuprizone demyelinating model, several, but not all, oligodendrocytes of the corpus callosum express TrkA and p75NTR [51, 52]. NGF exhibits sparsely scattered immunoreactivity with oligodendrocytes (data not shown). Our results bear potential clinical implications for EAE and MS, both conditions characterized by inflammatory demyelination and loss of oligodendrocytes.

As for the other glial cell population, there is observed scattered immunoreactivity with the fibrous astrocytes within the glial scar but not in the NAWM. Particularly for NGF, immunoreactivity is located only in astrocytic processes, found in our study for the first time. TrkA and p75NTR are primary located around the cell bodies. It is of note that not all the astrocytes express the molecules under investigation. These results are supported in part by other studies reporting high expression of TrkA and a lower of p75NTR in radial astrocytes in rats with EAE. Their role on astrocytes have been associated with the migration of the neural precursor cells in the spinal cord while in chronic lesions of patients with MS, there is limited expression of TrkA and p75NTR on astrocytes [29, 53]. Regarding the effect of NGF on astrocytes, it has been shown that in epilepsy model, it can limit their proliferation through p75NTR [54]. Based on our results, we assume that the polarized expression of NGF in the processes and the receptors mostly in the cell body act in a paracrine way on the astrocytes of the glial scar influencing their proliferation. The role of astrocytes forming the glial scar is still not fully understood. On the one hand, they are believed to act destructively by mediating cytotoxicity via the production of cytotoxic metabolites, reactive oxygen species, etc., which leads to neuronal loss and degeneration [55, 56]. However, emerging notion supports their neuroprotective function mediated by neurotrophin production and signaling [57–59]. Based on our observation, it may be reasonable to assume that higher TrkA expression favors astrocyte survival and proliferation to support and protect the neurons, whereas p75NTR dominance limits the relative number of autoreactive astrocytes.

Our results show that there is no expression of the NGF, TrkA, and p75NTR on pro-inflammatory M1 phenotype microglia (iNOS^+^ cells). In contrast, we show for the first time that NGF, TrkA, and p75NTR co-localize with the anti-inflammatory M2 phenotype microglia (Arginase1^+^ cells) in the spinal cord during the EAE course. M2 microglia is regulated by NGF and its action via the high-affinity TrkA/p75NTR system. Microglia cultures were shown to produce in vitro the mRNA of NGF, BDNF, and NT-3 but not the proteins [60]. In MS lesions, p75NTR expression has been observed without distinguishing the microglial cell phenotype [61]. In addition, Barouch et al., in 2002, refer the expression of NGF in microglial cells in an axotomized optic nerve [8]. Our results propose anti-inflammatory and neuroprotective effects of M2 cells by maintaining neuronal function intact, a finding supported by the observation that NGF through p75NTR can suppress the expression of MHCII on microglial cells [62].

Analysis of the role of the NGF system expression, in cells of the immune system, showed the presence of these three molecules in macrophages (Mac-3^+^ cells). Our observation is made for the first time in the EAE model in high percentage. In studies employing human peripheral blood monocytes, the results are unclear as expression of NGF, TrkA, and p75NTR is reported in monocytes but not in macrophages and in a macrophage subpopulation isolated from patients with osteoarthritis [63–66]. The question that derives is whether macrophages synthesize and secrete NGF. Our results of NGF localized inside the macrophages could possibly be explained by its paracrine action meaning that it is taken up by cells through the TrkA/p75NTR receptor complex or that it is located in cells or/and structures that are phagocytosed by macrophages. Our observation is partially supported by a study where it has been shown that both the NGF-mRNA and the NGF protein were detected in macrophages isolated from patient with osteoarthritis [66], whereas the NGF-mRNA was also found in cultured mouse macrophages [67]. In general, it is supported that the neurotrophic factor NGF regulates in a paracrine way the migration of monocytes/macrophages into the CNS through the BBB [68].

It is noteworthy that while the pathophysiology of our model is mediated by T lymphocytes, neither NGF synthesis nor TrkA and p75NTR expression was observed in co-localization with the CD3 marker. At first glance, this is surprising primarily due to studies where it is indicated that both MBP-activated T lymphocytes and CD4^+^ cells, both in humans and rodents, produce the NGF and TrkA, whereas for p75NTR, there are limited references [69]. Low expression of NGF in CD4^+^ cells or CD3^+^ cells has been reported [70, 71]. Other studies failed to report co-localization of CD3 with TrkA and p75NTR in chronic lesions of MS patients or NGF expression in T lymphocytes in axotomized optic nerve [8, 53]. Several studies indicate the anti-inflammatory effect of NGF regarding the delayed onset and decreased manifestation of clinical symptoms of EAE [40, 72, 73]. Based on the previous data, we can hypothesize that the expression or not of NGF, TrkA, and p75NTR in the T lymphocytes may be related to various factors such as the phylogenetic differences of the studied organism (human or rodent), the disease (MS, passive or active EAE), and the T lymphocytes’ differentiation stage (naïve or activated), as well as the nature of the activating stimulus/antigen.

Finally, B cell analysis (B220^+^ cells) showed their capacity to synthesize NGF and express p75NTR but not TrkA. Our results show for the first time the co-expression with p75NTR through which the proliferation, differentiation, and production of antibodies can be regulated [74–76]. Also, there is the implication of the GC-like structures in the CNS (or B cell follicles) where B cells accumulate, whereas the existence and the role of these structures, both in MS and EAE, are controversial [77, 78]. It is believed that they preserve and renew the inflammatory conditions by activating T cells and producing auto-antibodies locally inside the CNS, avoiding the time-consuming peripheral trigger and also participating to the CNS neurodegeneration [79, 80]. So, NGF can act as an autocrine cytokine, a “neurokine”. p75NTR, in the absence of the TrkA co-receptor, could affect B cell migration via BBB, in which it has been described to have a crucial role, as well as limit the proliferation or production of auto-antibodies from resident B cells, as previously described for the astrocytes. In this way, NGF acts protectively in the context of protective autoimmunity, where the body develops mechanisms to cope with CNS damage by restricting and controlling the degeneration and/or promoting the regeneration [81, 82].

## Conclusion

By this study, both the neuroprotective and anti-inflammatory role of the NGF mediated by its two-receptor system, TrkA and p75NTR, are highlighted during the destructive inflammatory attack of the autoimmune response in our MOG-EAE model. NGF system is highly upregulated in the inflammatory loci during the EAE disease course, indicating its implication in the pathophysiological processes. Specifically, NGF system is expressed by the neurons in the grey matter and is upregulated in the acute and chronic phases in their attempt to survive. Also, they co-localize with the regenerative marker GAP-43 suggesting a possible role during regenerative processes. The expression of TrkA and p75NTR on oligodendrocytes is proved insufficient for their involvement in remyelination and raise the question of directing the NGF system to function adequately towards this direction. In addition, the different spatial expression of NGF system on astrocytes is another indication of its neuroprotective role by regulating the damage caused by astrogliosis. The anti-inflammatory property of the NGF system is attributed in part by its expression on M2 type microglia which can indirectly prevent the neurodegeneration. Despite the role of NGF, TrkA, and p75NTR on Mac-3^+^ cells being unclear, we speculate that the NGF system is involved in controlling their number and regulating their BBB migration. As far as it concerns, the immunomodulatory role of the NGF system seems to act on B cells and regulate their inflammatory functions by restricting the devastating inflammatory environment which ultimately aims at the CNS degeneration established especially in the chronic phase of the disease. The mechanisms by which all these events take place are still unclear and need further investigation. In addition, there are culprits that should be surpassed in order to reach the optimal results in neuroprotection, remyelination, and regeneration concomitant with the regulation of the immune response.

In this context, the efforts should be aimed to promote directly or indirectly the remyelination mechanism as well as to develop innovative techniques and/or pharmaceutical molecules that would contribute primarily to the survival, proliferation, and differentiation to myelinating oligodendrocytes and secondary to promote the axonal regeneration in pathological conditions. Nowadays, there is a growing body of evidence by studies using synthetic analogues that can easily cross the BBB due to their small molecular size and lipophilic properties and can mimic the beneficial functions of NGF without any possible side effects that should be taken into future consideration.

## Supplementary information

**Additional file 1: Figure S1.** The calculated p75NTR:TrkA ratio mRNA relative expression by qPCR analysis of brain (A) and spinal cord (B) during EAE course. (*p* < 0.05 (*), *p* < 0.01 (**), *p* < 0.001 (***), *p* < 0.0001 (****)).

**Additional file 2: Figure S2.** The calculated p75NTR:TrkA ratio protein relative expression by Western blotting analysis of brain (A) and spinal cord (B) during EAE course. (*p* < 0.05 (*), *p* < 0.01 (**), *p* < 0.001 (***), *p* < 0.0001 (****)).

## Data Availability

The datasets supporting the conclusions of this article are included within the article.

## Abbreviations

BAD: BCL2-associated agonist of cell death protein
BDNF: Brain-derived neurotrophic factor
cDNA: Complementary DNA
CFA: Complete Freund’s adjuvant
CNPase: 2′,3′-Cyclic-nucleotide 3′-phosphodiesterase
CNS: Central nervous system
CREB: cAMP response element binding protein
DAB: 3,3′-Diaminobenzidine
DAG: Diacylglycerol
EAE: Experimental autoimmune encephalomyelitis
EDTA: Ethylenediaminetetraacetic acid
EGTA: Egtazic acid
FBS: Fetal bovine serum
GAP-43: Growth-associated protein 43
GFAP: Glial fibrillary acidic protein
IFA: Incomplete Freund’s adjuvant
iNOS2: Inducible nitric oxide synthase (isoform 2)
IP3: Inositol trisphosphate
LBF: Luxol Fast Blue
MOG: Oligodendrocyte myelin glycoprotein
mRNA: Messenger RNA
MS: Multiple sclerosis
NeuN: Neuronal nuclei
NFκ-B: Nuclear factor kappa-light-chain-enhancer of activated B cells
NGF: Nerve growth factor
NGS: Normal goat serum
p75NTR: Pan-neurotrophin receptor p75
PBS: Phosphate-buffered saline
PFA: Paraformaldehyde
PI3K: Phosphoinositide 3-kinase
PLCγ: Phosphoinositide phospholipase C
PVDF: Polyvinylidene difluoride
qPCR: Semi-quantitative real-time PCR
SDS-PAGE: Sodium dodecyl sulfate polyacrylamide gel electrophoresis
SMI-32: Anti-neurofilament H non-phosphorylated (clone 32)
TBS: Tris-buffered saline
TBST: TBS supplemented with 0.1% Tween-20
TrkA: Tropomyosin receptor kinase A
TrkB: Tropomyosin receptor kinase B
WB: Western blot

## References

[1] Frank-Cannon TC, Alto LT, McAlpine FE, Tansey MG (2009). Does neuroinflammation fan the flame in neurodegenerative diseases?. Mol Neurodegener.

[2] Hohlfeld R, Kerschensteiner M, Stadelmann C, Lassmann H, Wekerle H (2006). The neuroprotective effect of inflammation: implications for the therapy of multiple sclerosis. Neurol Sci.

[3] Kigerl KA, Gensel JC, Ankeny DP, Alexander JK, Donnelly DJ, Popovich PG (2009). Identification of two distinct macrophage subsets with divergent effects causing either neurotoxicity or regeneration in the injured mouse spinal cord. J Neurosci.

[4] Moalem G, Leibowitz-Amit R, Yoles E, Mor F, Cohen IR, Schwartz M (1999). Autoimmune T cells protect neurons from secondary degeneration after central nervous system axotomy. Nat Med.

[5] Olsson T, Lidman O, Piehl F (2003). Harm or heal-divergent effects of autoimmune neuroinflammation?. Trends Immunol.

[6] Schwartz M, Deczkowska A (2016). Neurological disease as a failure of brain-immune crosstalk: the multiple faces of neuroinflammation. Trends Immunol.

[7] Schwartz M, Butovsky O, Kipnis J (2006). Does inflammation in an autoimmune disease differ from inflammation in neurodegenerative diseases? Possible implications for therapy. J NeuroImmune Pharmacol.

[8] Barouch R, Schwartz M (2002). Autoreactive T cells induce neurotrophin production by immune and neural cells in injured rat optic nerve: implications for protective autoimmunity. FASEB J.

[9] Schwartz M, Baruch K (2014). Breaking peripheral immune tolerance to CNS antigens in neurodegenerative diseases: boosting autoimmunity to fight-off chronic neuroinflammation. J Autoimmun.

[10] Shechter R, Miller O, Yovel G, Rosenzweig N, London A, Ruckh J, Kim K-W, Klein E, Kalchenko V, Bendel P (2013). Recruitment of beneficial M2 macrophages to injured spinal cord is orchestrated by remote brain choroid plexus. Immunity.

[11] Levi-Montalcini R, Skaper SD, Dal Toso R, Petrelli L, Leon A (1996). Nerve growth factor: from neurotrophin to neurokine. Trends Neurosci.

[12] Bibel M, Barde YA (2000). Neurotrophins: key regulators of cell fate and cell shape in the vertebrate nervous system. Genes Dev.

[13] Barbacid M (1995). Neurotrophic factors and their receptors. Curr Opin Cell Biol.

[14] Patapoutian A, Reichardt LF (2001). Trk receptors: mediators of neurotrophin action. Curr Opin Neurobiol.

[15] Chao MV (1994). The p75 neurotrophin receptor. J Neurobiol.

[16] Barker PA (2004). p75NTR is positively promiscuous: novel partners and new insights. Neuron.

[17] Chao MV (2003). Neurotrophins and their receptors: a convergence point for many signalling pathways. Nat Rev Neurosci.

[18] Chao MV, Hempstead BL (1995). p75 and Trk: a two-receptor system. Trends Neurosci.

[19] Friedman WJ, Greene LA (1999). Neurotrophin signaling via Trks and p75. Exp Cell Res.

[20] Greene LA, Kaplan DR (1995). Early events in neurotrophin signalling via Trk and p75 receptors. Curr Opin Neurobiol.

[21] Huang EJ, Reichardt LF (2003). Trk receptors: roles in neuronal signal transduction. Annu Rev Biochem.

[22] Kaplan DR, Miller FD (2000). Neurotrophin signal transduction in the nervous system. Curr Opin Neurobiol.

[23] Levy YS, Gilgun-Sherki Y, Melamed E, Offen D (2005). Therapeutic potential of neurotrophic factors in neurodegenerative diseases. BioDrugs.

[24] Reichardt LF (2006). Neurotrophin-regulated signalling pathways. Philos Trans R Soc Lond Ser B Biol Sci.

[25] Calza L, Giardino L, Pozza M, Micera A, Aloe L (1997). Time-course changes of nerve growth factor, corticotropin-releasing hormone, and nitric oxide synthase isoforms and their possible role in the development of inflammatory response in experimental allergic encephalomyelitis. Proc Natl Acad Sci U S A.

[26] Copray S, Kust B, Emmer B, Lin MY, Liem R, Amor S, de Vries H, Floris S, Boddeke E (2004). Deficient p75 low-affinity neurotrophin receptor expression exacerbates experimental allergic encephalomyelitis in C57/BL6 mice. J Neuroimmunol.

[27] De Simone R, Micera A, Tirassa P, Aloe L (1996). mRNA for NGF and p75 in the central nervous system of rats affected by experimental allergic encephalomyelitis. Neuropathol Appl Neurobiol.

[28] Nataf S, Naveilhan P, Sindji L, Darcy F, Brachet P, Montero-Menei CN (1998). Low affinity NGF receptor expression in the central nervous system during experimental allergic encephalomyelitis. J Neurosci Res.

[29] Oderfeld-Nowak B, Zaremba M, Lipkowski AW, Kwiatkowska-Patzer B, Triaca V, Aloe L (2003). High-affinity NGF receptor in the rat spinal cord during acute and chronic phases of experimental autoimmune encephalomyelitis: a possible functional significance. Arch Ital Biol.

[30] Oderfeld-Nowak B, Zaremba M, Micera A, Aloe L (2001). The upregulation of nerve growth factor receptors in reactive astrocytes of rat spinal cord during experimental autoimmune encephalomyelitis. Neurosci Lett.

[31] Soilu-Hanninen M, Epa R, Shipham K, Butzkueven H, Bucci T, Barrett G, Bartlett PF, Kilpatrick TJ (2000). Treatment of experimental autoimmune encephalomyelitis with antisense oligonucleotides against the low affinity neurotrophin receptor. J Neurosci Res.

[32] Kesidou E, Touloumi O, Lagoudaki R, Nousiopoulou E, Theotokis P, Poulatsidou KN, Boziki M, Kofidou E, Delivanoglou N, Minti F (2017). Humoral response in experimental autoimmune encephalomyelitis targets neural precursor cells in the central nervous system of naive rodents. J Neuroinflammation.

[33] Theotokis P, Touloumi O, Lagoudaki R, Nousiopoulou E, Kesidou E, Siafis S, Tselios T, Lourbopoulos A, Karacostas D, Grigoriadis N, Simeonidou C (2016). Nogo receptor complex expression dynamics in the inflammatory foci of central nervous system experimental autoimmune demyelination. J Neuroinflammation.

[34] Lassmann H, Bradl M (2017). Multiple sclerosis: experimental models and reality. Acta Neuropathol.

[35] Ransohoff RM (2012). Animal models of multiple sclerosis: the good, the bad and the bottom line. Nat Neurosci.

[36] Robinson AP, Harp CT, Noronha A, Miller SD (2014). The experimental autoimmune encephalomyelitis (EAE) model of MS: utility for understanding disease pathophysiology and treatment. Handb Clin Neurol.

[37] Wehrman T, He X, Raab B, Dukipatti A, Blau H, Garcia KC (2007). Structural and mechanistic insights into nerve growth factor interactions with the TrkA and p75 receptors. Neuron.

[38] Anastasia A, Barker PA, Chao MV, Hempstead BL (2015). Detection of p75NTR trimers: implications for receptor stoichiometry and activation. J Neurosci.

[39] He XL, Garcia KC (2004). Structure of nerve growth factor complexed with the shared neurotrophin receptor p75. Science.

[40] Micera A, Properzi F, Triaca V, Aloe L (2000). Nerve growth factor antibody exacerbates neuropathological signs of experimental allergic encephalomyelitis in adult Lewis rats. J Neuroimmunol.

[41] Verge VM, Tetzlaff W, Bisby MA, Richardson PM (1990). Influence of nerve growth factor on neurofilament gene expression in mature primary sensory neurons. J Neurosci.

[42] Chen Y, Zeng J, Cen L, Wang X, Yao G, Wang W, Qi W, Kong K (2009). Multiple roles of the p75 neurotrophin receptor in the nervous system. J Int Med Res.

[43] Culmsee C, Gerling N, Lehmann M, Nikolova-Karakashian M, Prehn JH, Mattson MP, Krieglstein J (2002). Nerve growth factor survival signaling in cultured hippocampal neurons is mediated through TrkA and requires the common neurotrophin receptor P75. Neuroscience.

[44] DeFreitas MF, McQuillen PS, Shatz CJ (2001). A novel p75NTR signaling pathway promotes survival, not death, of immunopurified neocortical subplate neurons. J Neurosci.

[45] Lad SP, Peterson DA, Bradshaw RA, Neet KE (2003). Individual and combined effects of TrkA and p75NTR nerve growth factor receptors. A role for the high affinity receptor site. J Biol Chem.

[46] Cai D, Shen Y, De Bellard M, Tang S, Filbin MT (1999). Prior exposure to neurotrophins blocks inhibition of axonal regeneration by MAG and myelin via a cAMP-dependent mechanism. Neuron.

[47] Domeniconi M, Zampieri N, Spencer T, Hilaire M, Mellado W, Chao MV, Filbin MT (2005). MAG induces regulated intramembrane proteolysis of the p75 neurotrophin receptor to inhibit neurite outgrowth. Neuron.

[48] Fujita Y, Yamashita T (2014). Axon growth inhibition by RhoA/ROCK in the central nervous system. Front Neurosci.

[49] Podbielska M, Banik NL, Kurowska E, Hogan EL (2013). Myelin recovery in multiple sclerosis: the challenge of remyelination. Brain Sci.

[50] Yamashita T, Tohyama M (2003). The p75 receptor acts as a displacement factor that releases Rho from Rho-GDI. Nat Neurosci.

[51] Bonetto G, Charalampopoulos I, Gravanis A, Karagogeos D (2017). The novel synthetic microneurotrophin BNN27 protects mature oligodendrocytes against cuprizone-induced death, through the NGF receptor TrkA. Glia.

[52] Casaccia-Bonnefil P, Carter BD, Dobrowsky RT, Chao MV (1996). Death of oligodendrocytes mediated by the interaction of nerve growth factor with its receptor p75. Nature.

[53] Valdo P, Stegagno C, Mazzucco S, Zuliani E, Zanusso G, Moretto G, Raine CS, Bonetti B (2002). Enhanced expression of NGF receptors in multiple sclerosis lesions. J Neuropathol Exp Neurol.

[54] Cragnolini AB, Huang Y, Gokina P, Friedman WJ (2009). Nerve growth factor attenuates proliferation of astrocytes via the p75 neurotrophin receptor. Glia.

[55] Brosnan CF, Raine CS (2013). The astrocyte in multiple sclerosis revisited. Glia.

[56] Rossi D, Volterra A (2009). Astrocytic dysfunction: insights on the role in neurodegeneration. Brain Res Bull.

[57] Hamby ME, Sofroniew MV (2010). Reactive astrocytes as therapeutic targets for CNS disorders. Neurotherapeutics.

[58] Li K, Li J, Zheng J, Qin S (2019). Reactive astrocytes in neurodegenerative diseases. Aging Dis.

[59] Raposo C, Schwartz M (2014). Glial scar and immune cell involvement in tissue remodeling and repair following acute CNS injuries. Glia.

[60] Elkabes S, DiCicco-Bloom EM, Black IB (1996). Brain microglia/macrophages express neurotrophins that selectively regulate microglial proliferation and function. J Neurosci.

[61] Dowling P, Ming X, Raval S, Husar W, Casaccia-Bonnefil P, Chao M, Cook S, Blumberg B (1999). Up-regulated p75NTR neurotrophin receptor on glial cells in MS plaques. Neurology.

[62] Neumann H, Misgeld T, Matsumuro K, Wekerle H (1998). Neurotrophins inhibit major histocompatibility class II inducibility of microglia: involvement of the p75 neurotrophin receptor. Proc Natl Acad Sci.

[63] Caroleo MC, Costa N, Bracci-Laudiero L, Aloe L (2001). Human monocyte/macrophages activate by exposure to LPS overexpress NGF and NGF receptors. J Neuroimmunol.

[64] Ehrhard PB, Ganter U, Stalder A, Bauer J, Otten U (1993). Expression of functional trk protooncogene in human monocytes. Proc Natl Acad Sci U S A.

[65] Prencipe G, Minnone G, Strippoli R, De Pasquale L, Petrini S, Caiello I, Manni L, De Benedetti F, Bracci-Laudiero L (2014). Nerve growth factor downregulates inflammatory response in human monocytes through TrkA. J Immunol.

[66] Takano S, Uchida K, Inoue G, Miyagi M, Aikawa J, Iwase D, Iwabuchi K, Matsumoto T, Satoh M, Mukai M (2017). Nerve growth factor regulation and production by macrophages in osteoarthritic synovium. Clin Exp Immunol.

[67] Barouch R, Kazimirsky G, Appel E, Brodie C (2001). Nerve growth factor regulates TNF-alpha production in mouse macrophages via MAP kinase activation. J Leukoc Biol.

[68] Linker R, Gold R, Luhder F (2009). Function of neurotrophic factors beyond the nervous system: inflammation and autoimmune demyelination. Crit Rev Immunol.

[69] Ehrhard PB, Erb P, Graumann U, Otten U (1993). Expression of nerve growth factor and nerve growth factor receptor tyrosine kinase Trk in activated CD4-positive T-cell clones. Proc Natl Acad Sci U S A.

[70] Lambiase A, Bracci-Laudiero L, Bonini S, Starace G, D'Elios MM, De Carli M, Aloe L (1997). Human CD4+ T cell clones produce and release nerve growth factor and express high-affinity nerve growth factor receptors. J Allergy Clin Immunol.

[71] Moalem G, Gdalyahu A, Shani Y, Otten U, Lazarovici P, Cohen IR, Schwartz M (2000). Production of neurotrophins by activated T cells: implications for neuroprotective autoimmunity. J Autoimmun.

[72] Arredondo LR, Deng C, Ratts RB, Lovett-Racke AE, Holtzman DM, Racke MK (2001). Role of nerve growth factor in experimental autoimmune encephalomyelitis. Eur J Immunol.

[73] Villoslada P, Hauser SL, Bartke I, Unger J, Heald N, Rosenberg D, Cheung SW, Mobley WC, Fisher S, Genain CP (2000). Human nerve growth factor protects common marmosets against autoimmune encephalomyelitis by switching the balance of T helper cell type 1 and 2 cytokines within the central nervous system. J Exp Med.

[74] Blauth K, Owens GP, Bennett JL (2015). The ins and outs of B cells in multiple sclerosis. Front Immunol.

[75] Magliozzi R, Howell O, Vora A, Serafini B, Nicholas R, Puopolo M, Reynolds R, Aloisi F (2007). Meningeal B-cell follicles in secondary progressive multiple sclerosis associate with early onset of disease and severe cortical pathology. Brain.

[76] Pikor N, Gommerman JL (2012). B cells in MS: why, where and how?. Mult Scler Relat Disord.

[77] Batoulis H, Wunsch M, Birkenheier J, Rottlaender A, Gorboulev V, Kuerten S (2015). Central nervous system infiltrates are characterized by features of ongoing B cell-related immune activity in MP4-induced experimental autoimmune encephalomyelitis. Clin Immunol.

[78] Comabella M, Canto E, Nurtdinov R, Rio J, Villar LM, Picon C, Castillo J, Fissolo N, Aymerich X, Auger C (2016). MRI phenotypes with high neurodegeneration are associated with peripheral blood B-cell changes. Hum Mol Genet.

[79] Correale J, Marrodan M, Ysrraelit MC. Mechanisms of Neurodegeneration and Axonal Dysfunction in Progressive Multiple Sclerosis. Biomedicines. 2019;7(1). 10.3390/biomedicines7010014 PMC6466454 30791637

[80] Hausser-Kinzel S, Weber MS (2019). The role of B cells and antibodies in multiple sclerosis, neuromyelitis optica, and related disorders. Front Immunol.

[81] Cohen IR, Schwartz M (1999). Autoimmune maintenance and neuroprotection of the central nervous system. J Neuroimmunol.

[82] Schwartz M, Raposo C (2014). Protective autoimmunity: a unifying model for the immune network involved in CNS repair. Neuroscientist.

