# Supplementary figures and images for: Spatio-temporal expression profile of NGF and the two-receptor system, TrkA and p75NTR, in experimental autoimmune encephalomyelitis
Source: J Neuroinflammation. 2020 Jan 29;17:41. doi: 10.1186/s12974-020-1708-9 (PMC6990493; doi:10.1186/s12974-020-1708-9)

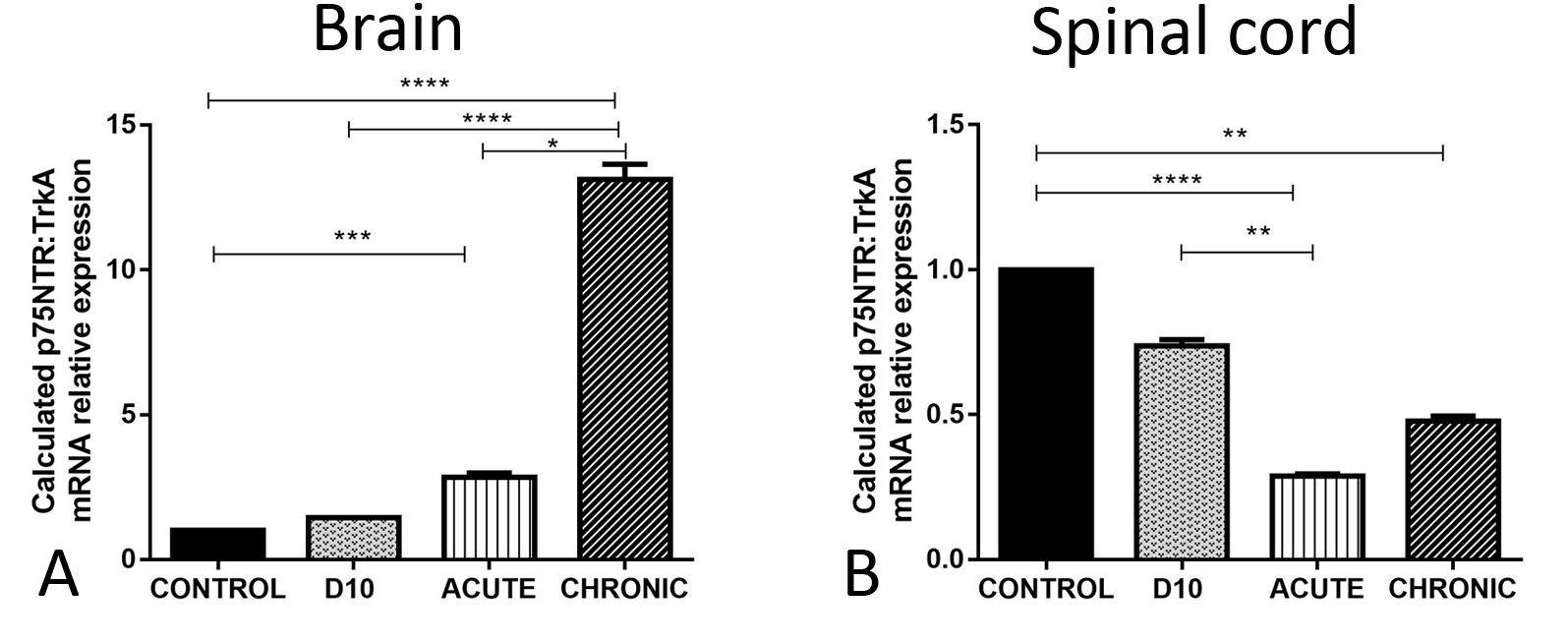

Supplement: Supplementary file 1 — Additional file 1: Figure S1. The calculated p75NTR:TrkA ratio mRNA relative expression by qPCR analysis of brain (A) and spinal cord (B) during EAE course. (p < 0.05 (*), p < 0.01 (**), p < 0.001 (***), p < 0.0001 (****)). [file 12974_2020_1708_MOESM1_ESM.jpg]

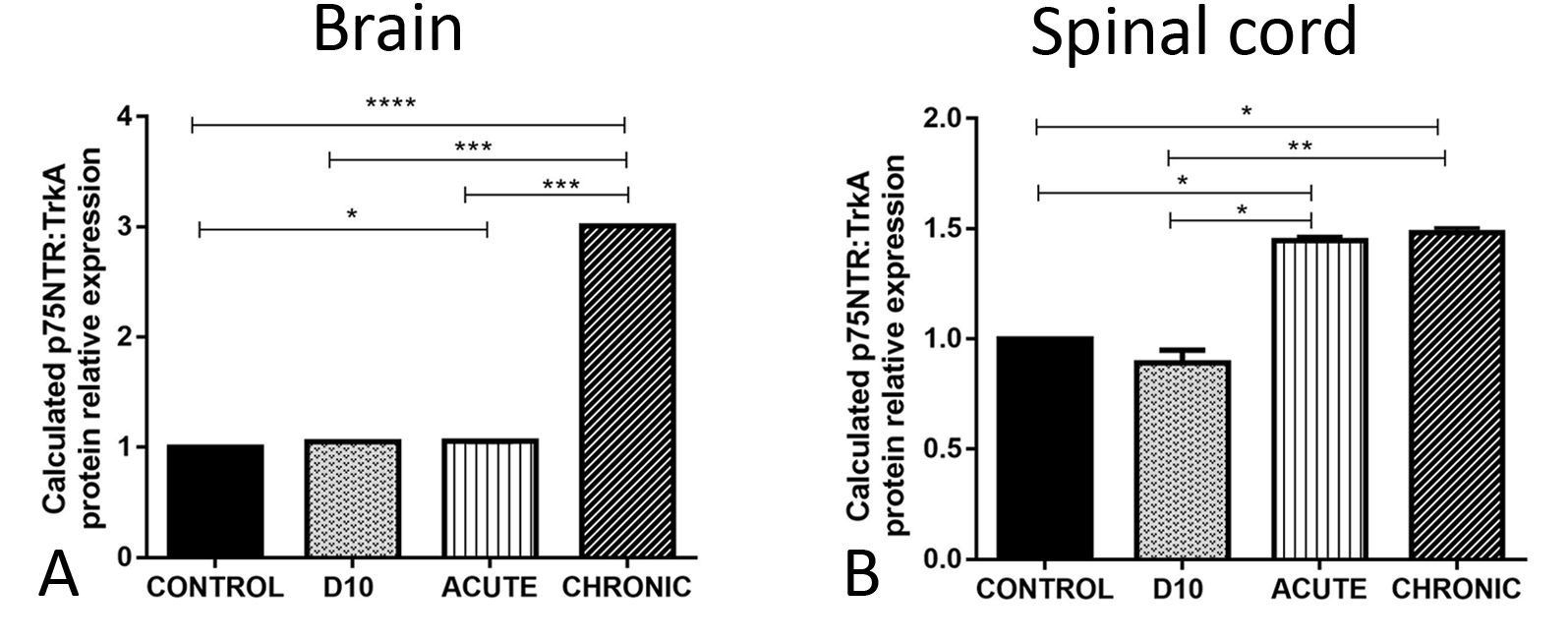

Supplement: Supplementary file 2 — Additional file 2: Figure S2. The calculated p75NTR:TrkA ratio protein relative expression by Western blotting analysis of brain (A) and spinal cord (B) during EAE course. (p < 0.05 (*), p < 0.01 (**), p < 0.001 (***), p < 0.0001 (****)). [file 12974_2020_1708_MOESM2_ESM.jpg]
